# Effects of blood flow restriction on muscle size and gene expression in muscle during immobilization: A pilot study

**DOI:** 10.14814/phy2.14516

**Published:** 2020-07-29

**Authors:** Saori Kakehi, Yoshifumi Tamura, Atsushi Kubota, Kageumi Takeno, Minako Kawaguchi, Keishoku Sakuraba, Ryuzo Kawamori, Hirotaka Watada

**Affiliations:** ^1^ Department of Metabolism & Endocrinology Juntendo University Graduate School of Medicine Tokyo Japan; ^2^ Sportology Center Juntendo University Graduate School of Medicine Tokyo Japan; ^3^ Department of Sports Medicine Juntendo University Graduate School of Health and Sports Science Chiba Japan; ^4^ Center for Therapeutic Innovations in Diabetes Juntendo University Graduate School of Medicine Tokyo Japan; ^5^ Center for Molecular Diabetology Juntendo University Graduate School of Medicine Tokyo Japan

**Keywords:** *MuRF1*, muscle atrophy, muscle disuse

## Abstract

**Purpose:**

Muscle mass is known to rapidly decrease with muscle disuse. Previous reports suggest that repetitive blood flow restriction (BFR) mitigates the reduction of muscle mass with disuse. However, the effects of BFR on muscle atrophy and gene expression levels in muscle during cast immobilization have not been clarified.

**Methods:**

To investigate the effect of BFR on muscle atrophy and gene expression levels during cast immobilization in humans, we recruited 10 healthy males who were randomly divided into the control and BFR treatment groups. All subjects were immobilized with a cast for 14 days. BFR treatment was conducted only in the BFR group. We evaluated cross sectional area (CSA) of thigh muscles by magnetic resonance imaging before and 14 days after cast immobilization. A percutaneous biopsy of the vastus lateralis muscle (VL) was performed before and 1, 7, and 14 days after cast immobilization. Expression of genes related to muscle atrophy and synthesis were evaluated using real‐time PCR.

**Results:**

The CSA of the VL and the thigh flexor muscles were significantly decreased in both groups; however, percent decrease in CSA was significantly smaller in the BFR group compared with the control group. In two‐way repeated ANOVA analysis, the time × treatment interaction in gene expression of the muscle‐specific ubiquitin ligases muscle ring finger 1 (*MuRF1*) was significant, and elevated MURF1 expression level by cast immobilization was seemed to be suppressed by the BFR treatment.

**Conclusion:**

BFR treatment may prevent reduced VL and thigh flexor muscles and increased *MuRF1* expression level during cast immobilization. Further study is required to confirm these results.

## Graphical abstract

Previous reports suggest that repetitive blood flow restriction (BFR) mitigates the reduction of muscle mass with disuse. However, the effects of BFR on gene expression levels in muscle during cast immobilization have not been clarified. Our data suggested that BFR treatment might alter the expression of key genes related to muscle atrophy and protein synthesis, thereby mitigating the reduction in muscle mass associated with cast immobilization in healthy males.

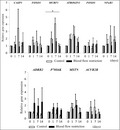

## 1 INTRODUCTION

Muscle mass and strength rapidly decrease with muscle disuse, such as during bed rest and cast immobilization (Brooks & Myburgh, 2014; Wall, Dirks, & van Loon, 2013; Zhang, Chen, & Fan, 2007). In the clinical setting, muscle disuse is frequently observed after falls, bone fractures, and apoplexy in elderly individuals. It is also observed in young people after injuries. Decreased muscle mass and strength are reported to be independent risk factors for falls, reduced quality of life, and death in the elderly (Kalyani, Corriere, & Ferrucci, 2014; Legrand et al., 2014) and impede early return from injury among athletes. Thus, preventing the loss of muscle mass and strength during disuse is important in various situations. To establish an efficient method for preventing muscle atrophy, it is important to understand the molecular mechanisms of disuse‐induced muscle atrophy.

As the first step to developing methods for preventing the loss of muscle mass, repetitive blood flow restriction (BFR) method was developed and many studies were performed. Recent meta‐analysis indicated that low‐load training with BFR and walking with BFR is an effective method for muscle hypertrophy and strength gains in older populations (Centner, Wiegel, Gollhofer, & Konig, 2019). On the other hand, we also demonstrated that BFR without strength training may mitigate muscle weakness and atrophy induced by 14 days of cast immobilization (CI) with non‐weight bearing (NWB) (Kubota, Sakuraba, Sawaki, Sumide, & Tamura, 2008). In the study, while thigh circumference and muscle strength decreased after 14 days of CI and NWB, BFR treatment mitigated these changes. BFR had a more pronounced preventive effect than isometric training. Thus, the BFR treatment itself has potential to prevent muscle atrophy induced by disuse; however, it was not proved by imaging devices. In addition, the molecular mechanisms of preventive effect of BFR on reduced muscle mass during CI and NWB have not been clarified yet.

Skeletal muscle mass is maintained through a precise balance between anabolic (protein synthesis) and catabolic (protein degradation) states (Wall et al., 2013). It has been suggested that short‐term disuse (less than approximately 10 days) induces muscle atrophy via protein breakdown and decreased protein synthesis (Marimuthu, Murton, & Greenhaff, 2011; Urso, Scrimgeour, Chen, Thompson, & Clarkson, 2006; Wall et al., 2013, 2015). However, with more prolonged disuse, only decreased protein synthesis is observed (Bodine et al., 2001; Wall et al., 2013). Although many genes are involved in muscle atrophy in each period, two muscle‐specific ubiquitin ligases, *Atrogin 1* and muscle ring finger 1 (*MuRF1*), (Bodine et al., 2001; Brooks & Myburgh, 2014; Wall et al., 2013) play especially important roles in muscle atrophy. Previous studies suggest that protein breakdown and related gene expression in muscle, including *Atrogin 1* and *MuRF1*, are upregulated approximately 1–7 days after muscle disuse and recover to baseline values at least 14 days after disuse (Abadi et al., 2009; Bodine et al., 2001; Wall et al., 2016). Thus, the BFR treatment may suppress protein breakdown related gene expression, including *Atrogin 1* and *MuRF1*, during CI and NWB, then prevent muscle atrophy.

Given this context, we aimed to clarify the effect of BFR treatment on muscle size by magnetic resonance imaging (MRI) and gene expression levels related to muscle atrophy during CI and NWB as a pilot study.

## 2 METHODS

### 2.1 Subjects and study design

**FIGURE 1:**
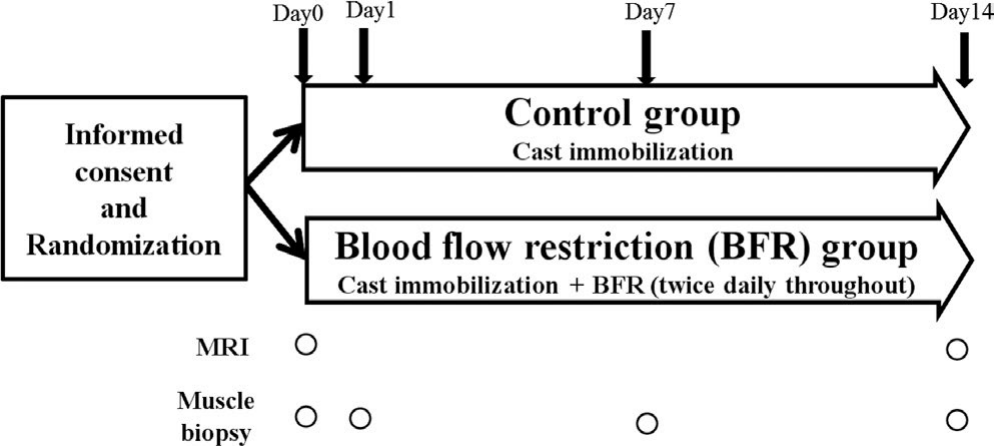
Study protocol. MRI, magnetic resonance imaging

Subjects were 10 healthy untrained males with no history of injuries to the lower extremities or serious medical complaints. The study protocol is outlined in Figure 1. Subjects were prohibited from exercise 3 days before the study. Subjects were divided randomly into two groups: subjects who received CI only (control group) and subjects who received CI and repetitive BFR (BFR group). CI was conducted by fixing the left ankle and knee joints in neutral positions with a cast. Subjects were instructed to always walk using crutches for NWB over 14 days. BFR was conducted by restricting blood flow to the lower left extremity by compressing the proximal end of the thigh using a tourniquet that was 77 mm in width and 770 mm in length (MIZUHO Co. Ltd., Tokyo, Japan) with 200 mm Hg of compressive force as described previously (Kubota et al., 2008). A single set consisted of 5 min of BFR followed by 3 min of rest (release of compression). Each subject underwent five sets twice a day (morning and afternoon) for 14 days. Before and after the 14‐day intervention, we evaluated cross sectional area (CSA) of the thigh muscle using MRI. A percutaneous biopsy of the right vastus lateralis muscle was performed with local anesthesia before the intervention. At 1, 7, and 14 days after the intervention, muscle biopsy of the left vastus lateralis muscle was performed. The right side was used for biopsy at baseline to avoid the effects of acute inflammation induced by biopsy on the immobilized side. The muscle biopsy samples were flash‐frozen in liquid nitrogen after trimming of visible adipose tissue and stored at −80°C for real‐time PCR analysis (Kakehi et al., 2016; Kawaguchi et al., 2014). All participants gave written informed consent for study participation. The study was approved by the ethics committee of Juntendo University and conducted in accordance with the principles outlined in the Declaration of Helsinki.

### 2.2 Measurement of thigh muscle cross sectional area measurement using magnetic resonance imaging

To determine changes in the area of thigh muscles, T1‐weighted MRI images were obtained with a 0.2‐T scanner (E‐scanXQ, Esaote). Sequence conditions were TR, 740 ms; TE, 18 ms; NEX, 2; slice thickness, 5 mm; and FOV, 170 mm. We evaluated CSA of thigh muscles on the medial side at 10 cm from the superior aspect of the patella. All MRI data were transferred to a computer and CSA was evaluated using NIH imaging software (National Institutes of Health, Bethesda, MD).

### 2.3 RNA isolation and assessment of mRNA levels for real‐time quantitative PCR

Frozen muscle samples were homogenized with 0.5 ml of TRIzol Reagent (Thermo Fisher Scientific) using TissueLyser (Qiagen). Total RNA from muscle samples was isolated using the RNeasy lipid Tissue mini kit (Qiagen). Using the High Capacity cDNA Reverse Transcription Kit (Thermo Fisher Scientific), 1 microgram of total RNA was reverse transcribed to cDNA. Real‐time PCR was performed using the protocols and detection systems of the ABI Prism 7500 Fast Sequence Detection System (Thermo Fisher Scientific) (Kakehi et al., 2016; Kawaguchi et al., 2014). Primers were designed using Primer‐BLAST (NCBI). PCR products were detected using Fast SYBR^®^ Green Master Mix (Thermo Fisher Scientific). The −ΔΔCT method was used to calculate the relative expression ratio (2^−ΔΔCT^) based on the change in threshold values. Expression levels of the target genes were normalized with TATA‐Box binding protein (TBP) expression levels, which reflect standard endogenous expression. TBP expression was not significantly different between any time points. All samples and standards were analyzed in duplicate.

In the present study, we evaluated serial changes in the expression levels of genes related to catabolism and anabolism. For example, levels of *Atrogin 1* and *MuRF1* are positively regulated by transcription factors Forkhead boxO 1 (FOXO1) and FOXO3. MuRF1 expression is positively regulated by nuclear factor kappa B (NFκB) (Bodine & Baehr, 2014; Brooks & Myburgh, 2014; Wall et al., 2013; Zhang et al., 2007). Caspase (CASP)‐3 activation is the initial step triggering accelerated muscle proteolysis during catabolic conditions (Du et al., 2004). On the other hand, β2 adrenergic receptor (ADRB2)‐PI3K‐AKT signaling induces protein synthesis through P70S6K activation and suppresses muscle protein degradation through FOXO1 and FOXO3 inactivation (Lynch & Ryall, 2008). In addition, myostatin (MSTN), a member of the TGFβ superfamily, is secreted by muscle and acts as via autocrine or paracrine signaling through the activin receptor type‐2B (ACVR2B) to reduce muscle mass (Brooks & Myburgh, 2014; Kollias & McDermott, 2008; Lee & McPherron, 2001; McPherron, Lawler, & Lee, 1997; McPherron & Lee, 2002; Reisz‐Porszasz et al., 2003). Thus, we investigated the expression levels of these genes.

### 2.4 Statistical analysis

All data are expressed as means ± *SD*. The CSA changes within each group and between the groups were analyzed using Student's *t*‐test. Since this study is preliminary with small number of subjects, we compared gene expression levels by several method. The gene expression level within the group was analyzed using one‐way ANOVA, followed by the Tukey‐Kramer post hoc test. We also performed two‐way repeated ANOVA to analyze the time × treatment interaction. A *p* value of less than 0.05 was considered to indicate a statistically significant difference.

## 3 RESULTS

### 3.1 Anthropometric characteristics

Mean age and BMI in the control group and the BFR group were 22.0 ± 0 and 21.4 ± 1.1 y.o., 23.1 ± 0.7 and 22.4 ± 2.6 kg/m^2^, respectively, and there were no statistical differences between the groups. In addition, 14 days of CI with NWB did not result in significant changes in BMI in each group (the control group; from 23.1 ± 0.7 to 23.3 ± 0.7 kg/m^2^, the BFR group; from 22.4 ± 2.6 to 22.3 ± 2.6 kg/m^2^).

### 3.2 Effects of CI with NWB and BFR on muscle CSA

**FIGURE 2:**
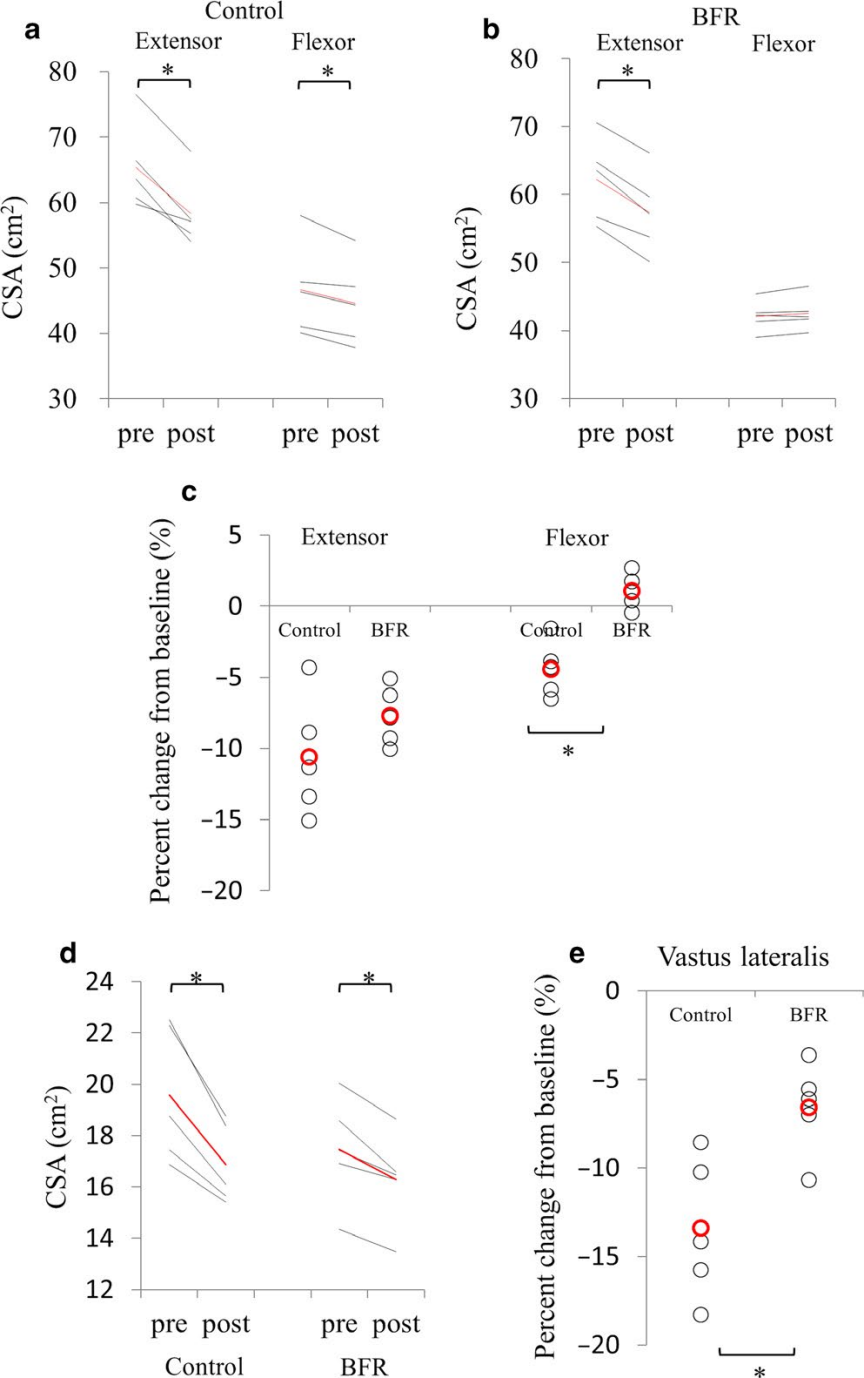
Changes in cross‐sectional area (CSA) of muscles in the control and blood flow restriction (BFR) groups. (a and b) Changes in CSA of thigh flexor and extensor muscles in the control group (a) and the BFR group (b). (c) Percent change in CSA in thigh flexor and extensor muscles. CSA compared with before CI is shown. (d and e) (e) Changes in CSA (d) and percent change (e) in the vastus lateralis muscle (a, b, d) Red line; average change. (c and e) Red circle; average change. *p < .05

As shown in Figure 2a, CI for 14 days significantly decreased CSA of the thigh extensor and flexor muscles in the control group. On the other hand, in the BFR group, we found a decrease in CSA of thigh extensor muscles after the intervention, but no significant change in CSA of thigh flexor muscles (Figure 2b). Accordingly, while percent decrease in CSA in extensor muscles was comparable between the groups, the control group had a significantly higher percent decrease in CSA in flexor muscles than the BFR group (Figure 2c). In addition, we specifically evaluated CSA changes in the vastus lateralis muscle, the site of muscle biopsies. As shown in Figure 2d, CSA of the vastus lateralis muscle was significantly decreased in both groups; however, percent decrease in CSA was significantly smaller in the BFR group compared with the control group (Figure 2e). These results indicate that BFR has a preventive effect on decrease in CSA induced by CI for some muscles.

### 3.3 Expression levels of genes related to skeletal muscle catabolism and anabolism

**FIGURE 3:**
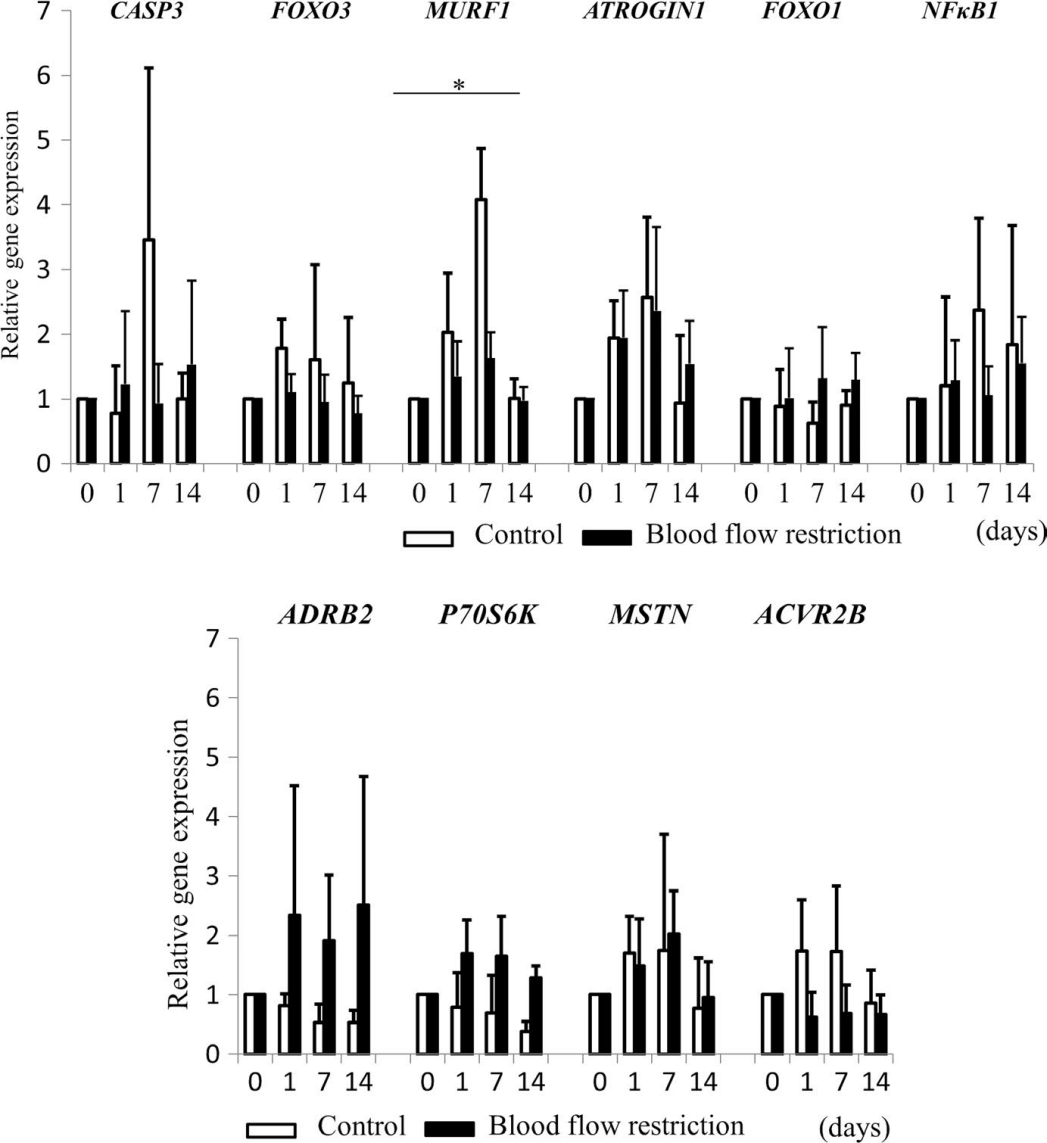
Changes in gene expression levels in the control and BFR groups. Quantification of mRNA levels relative to before CI (day 0). Relative expression levels were calculated by setting the expression level in the muscle before CI to 1 in each subject. Data are shown as means ± *SD* of five subjects in each group. Control group: white bar; BFR group: black bar, *p < .05 for two‐way repeated ANOVA

As shown in Figure 3, we evaluated serial changes in the expression levels of genes related to catabolism and anabolism during CI with NWB in both groups. Two‐way repeated ANOVA revealed significant time × treatment interaction in the gene expression of MURF1 (*p* < .05) (Figure 3). In addition, we preliminarily applied one‐way ANOVA analysis in each group and we only found significant differences on gene expression levels of ATROGIN 1 and MURF1 (*p* < .05), and post‐hoc analysis revealed that there were significant differences between day 0 and day 7 on ATROGIN 1, and day 7 and day 14 on ATROGIN 1 and MURF1 (Figure 3).

## 4 DISCUSSION

Our study demonstrated that the CSA of the thigh flexor and VL muscles were significantly decreased in both groups after CI with NWB; however, percent decrease in CSA was significantly smaller in the BFR group compared with the control group. In terms of gene expression levels, two‐way repeated ANOVA revealed significant time × treatment interaction in the gene expression of *MuRF1*.

Most of previous studies performed the BFR treatment with low‐load training or walking (Centner et al., 2019) and they were effective interventional approaches to stimulate muscle hypertrophy and strength gains in elderly. On the other hand, only our group used the BFR treatment without any training (Kubota et al., 2008) and the results suggested that the BFR treatment itself has potential to prevent muscle atrophy induced by disuse (Kubota et al., 2008); however, it was not proved by imaging devices. Thus, the present study firstly suggested the preventive effect of the BFR treatment without training on muscle atrophy during disuse. In addition, it is also suggested that the preventive effect of the BFR treatment on muscle atrophy depends on the position of skeletal muscle and we could collect muscle sample from effective site for BFR treatment. Thus, we could address the gene expression level associated with the preventive effect of BFR treatment on muscle atrophy during disuse.

During 14 days of limb immobilization, muscle atrophy was demonstrated to mostly occur in the first 7 days (White, Davies, & Brooksby, 1984). *Atrogin 1* and *MuRF1* expression as well as protein ubiquitination were higher in muscle during acute (2 days) to short‐term (approximately 10 days) disuse (Abadi et al., 2009; Bodine et al., 2001; Wall et al., 2016) and expression of these genes returned to baseline levels by 14 days after disuse. Concerning, the present study showed similar trends of gene expression level of *Atrogin 1* and *MuRF1* during disuse, and this study firstly showed that increased *MuRF1* expression during CI was mostly prevented by BFR treatment. Considering the fact that disuse induced muscle atrophy was prevented in *MuRF1* knockout mice (Bodine & Baehr, 2014; Legrand et al., 2014), reduced *MuRF1* expression might be involved in the preventive effect of BFR treatment on disuse induced muscle atrophy.

This study has several limitations. First, we only evaluated levels of gene expression in muscle; their physiological roles are still unclear. For example, previous reports revealed that 2–7 days of muscle disuse promotes muscle protein breakdown, but it is still unclear whether protein breakdown is promoted after 1 day of disuse in humans. In addition, we collected very small muscle samples to avoid tissue inflammation, thus we could not evaluate protein level of each molecule. Second, specific molecules we evaluated are regulated by factors other than expression level, such as protein modification (e.g., phosphorylation and acetylation). Therefore, gene expression levels do not necessarily indicate functional activity of the protein. Third, we used −ΔΔCT method to gene expression levels. This method required only small samples and was beneficial in the present study; however, we only had relative value for baseline expression level in each subject, which may make Type I error. Forth, the BFR protocol prevented disuse‐induced muscle atrophy in thigh flexor muscles, but not extensor muscles. It is still unclear why the effects of BFR treatment varied by muscle position. It is possible that our biopsy data may not be generalizable to muscles other than the vastus lateralis. Finally, this study is a pilot study performed in a small number of participants, thus, further study is required to confirm these results in a larger cohort. However, cast immobilization for 14 days is a very difficult intervention to be performed, thus our data may be valuable when a similar study is planned and performed in the future.

In conclusion, the BFR treatment may prevent reduced VL and thigh flexor muscles and increased *MuRF1* expression level during CI. Further study is required to confirm these results.

## CONFLICT OF INTEREST

All authors declare no conflict of interest.

## AUTHOR CONTRIBUTIONS

S.K. and Y.T. researched the data and contributed to study design, data collection, interpretation of results, wrote and edited the manuscript. A.K., K.T., and M.K participated in data collection, data analysis and contributed to the discussion. K.S., R.K., and H.W. contributed to study design, reviewed and edited the manuscript.

## ETHICAL STATEMENT

All study participants provided informed consent, and the study design was approved by an ethics review board.
